# Peptidic product derived from trypsin autolysis modulates insect digestive proteases and supports plant biochemical defense

**DOI:** 10.1002/ps.70579

**Published:** 2026-01-22

**Authors:** Daniel Guimarães Silva Paulo, Halina Schultz, Yaremis Beatriz Meriño Cabrera, Ian Lucas Batista Santos, Maria Clara Neves Gomes Rodrigues, Milena Godoi Lima, Geisiane Aparecida Mariano, Rafael Junior de Andrade, Humberto Josué de Oliveira Ramos, Maria Goreti de Almeida Oliveira

**Affiliations:** ^1^ Department of Entomology Federal University of Viçosa Viçosa Brazil; ^2^ Institute of Biotechnology Applied to Agriculture – BIOAGRO‐UFV Viçosa Brazil; ^3^ Metabolic Biosolutions Rio de Janeiro Brazil; ^4^ Departamento de Biologia Universidad del Atlantico Puerto Colombia Colombia; ^5^ Department of Biochemistry and Molecular Biology Federal University of Viçosa Viçosa Brazil; ^6^ Center of Analysis of Biomolecules, NuBioMol, Federal University of Viçosa Viçosa Brazil

**Keywords:** enzyme kinetics, fall armyworm, integrated pest management, lepidopterans, molecular docking, protease inhibitors

## Abstract

**BACKGROUND:**

*Spodoptera frugiperda*, commonly known as the fall armyworm, is a highly economically significant pest that affects various crops, resulting in substantial losses in productivity. Managing this pest primarily relies on chemical insecticides; however, the repeated development of resistance to these chemicals has rendered them less effective. Given this scenario, sustainable alternatives, such as the use of digestive enzyme inhibitors, have been investigated as potential tools in pest management.

**RESULTS:**

Molecular docking predicts a conserved, isoform‐spanning pose for GORE 3 on *S. frugiperda* trypsins, with occupancy of S1/S1′ and adjacent subsites and richer aromatic/hydrophobic contacts than the S1‐focused reference benzamidine. This binding topology aligns with the enzymology: GORE 3 acts as a competitive inhibitor (K_i_ = 4.00 mM) *versus* benzamidine (K_i_ = 1.64 mM), and IC_50_ determinations confirmed effective enzymatic blockade. *In vivo*, GORE 3 reduced larval body mass, extended the larval period, and produced mortality up to 46.66%. Nutritional and kinetic parameters were significantly affected, including increased K_M_ (reduced substrate affinity) and lower approximate digestibility at higher dietary levels. Despite these effects, neither the leaf area consumed nor the feeding preference index differed from those of the controls.

**CONCLUSION:**

Docking and bioassays collectively demonstrate that GORE 3 interacts with trypsin‐*like* proteases through a robust, multisite binding mode, resulting in competitive inhibition and measurable physiological effects in *S. frugiperda*. Although less potent than benzamidine based on K_i_ values, GORE3's isoform‐spanning interactions and its influence on digestive and developmental parameters highlight the relevance of studying protease‐inhibitor peptides as biochemical models for understanding lepidopteran digestive physiology. Future work should investigate peptide stability, compensatory digestive responses, and performance under diverse biological conditions to better clarify the biological significance of these interactions. © 2026 The Author(s). *Pest Management Science* published by John Wiley & Sons Ltd on behalf of Society of Chemical Industry.

## Graphical abstract

XXXX

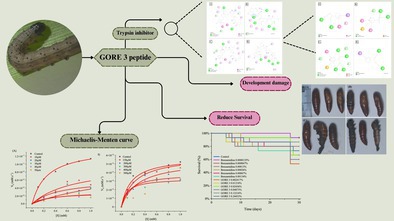

## 1 INTRODUCTION

Food security is a fundamental pillar of population growth, which drives the demand for food, making it necessary to develop new technologies to boost agricultural productivity. Given the substantial losses caused by diseases and pests, crop protection is crucial for ensuring food security. The use of agricultural pesticides remains one of the primary methods farmers use to prevent productivity losses. However, according to existing research, the excessive and improper use of pesticides has caused irreversible damage to the environment and public health through contamination of soil, water, and animals; loss of biodiversity; and the development of pest resistance to certain active ingredients in pesticides.

The fall armyworm, *Spodoptera frugiperda* (J.E. Smith) (Lepidoptera: Noctuidae), is one of the most important agricultural pests worldwide, causing significant yield losses in maize and other crops. The success of this pest is largely due to its remarkable adaptability and rapid territorial expansion, which have been further facilitated by global climate change.^1^ In Brazil, the use of chemical pesticides remains a crucial tool for controlling *S. frugiperda* in areas where it has developed resistance to B*t* corn.^2^ Reducing their use and employing integrated pest management programs can delay the evolution of insecticide resistance.^3^ In this context, there is an urgent demand for the development of sustainable management methods that are adaptable to production systems, such as biological control and integrated pest management approaches.^4^

Plants have developed defense adaptations that utilize natural compounds to block and slow the progression of stress. Plants produce and store protease inhibitors (PIs) in their fluids and tissues. For an effective pest control system, PIs with high stability under diverse physiological conditions are required. Several studies have demonstrated the potential of PIs in pest management.^5^, ^6^, ^7^, ^8^

Molecular docking has become an essential method in designing protease inhibitors, as it predicts how ligands interact with enzyme active sites and estimates their binding affinity. This technique helps identify compounds that can form strong interactions, such as hydrogen bonds, hydrophobic contacts, and ionic interactions, which are vital for effective inhibition. Recent studies have employed docking to investigate peptide inhibitors and bioinsecticidal proteins targeting insect proteases, providing valuable insights into pest control strategies.^1^, ^9^, ^10^ For research focused on controlling agricultural pests, the use of docking combined with molecular dynamics models has facilitated the assessment of how inhibitors interact with insect digestive proteases, the examination of the stability of these complexes, and the energy involved in their binding.^11^ This approach has been crucial in selecting molecules that are more specific and less prone to degradation, leading to the development of more effective, targeted bioinsecticides.

Using protease inhibitors can help manage pests, but insects have various ways to cope with the nutrient deficiency caused by these inhibitors, such as breaking them down, producing more proteases, or utilizing different types of proteases that are not affected by the inhibitors.^12^ Studies on the development of molecules with high specificity have been conducted to prevent their inactivation by the adaptive mechanisms of pest insects.^13^, ^14^

The GORE 3 peptide evaluated in this study was designed to have insecticidal potential, showing affinity for intestinal trypsin‐*like* proteases in lepidopterans, thereby disrupting key physiological processes involved in their development and survival. Studies in the literature report that insect trypsin‐*like* proteases exhibit a greater affinity for hydrophobic amino acids and preferentially cleave peptide bonds that contain basic amino acid residues, such as lysine and arginine.^15^, ^16^ To address this, we created GORE 3, which incorporates hydrophobic parts into its structure and terminates with tyrosine.

In this context, the present study aimed to evaluate the inhibitory capacity of the peptide GORE 3 on trypsin‐*like* enzyme activity in the midgut of *S. frugiperda* using *in silico*, *in vitro*, and *in vivo* approaches. These analyses contribute to the biochemical and physiological characterization of peptide–protease interactions in this agriculturally important species.

## 2 MATERIALS AND METHODS

### 2.1 Rearing of the caterpillars

Eggs of *S. frugiperda* used to initiate the trials were obtained from an established colony maintained at the Insect Rearing Laboratory, Department of Biochemistry and Molecular Biology, Federal University of Viçosa. After the eggs hatched, the caterpillars were individualized and fed an artificial diet within trays divided into cells. The artificial diet used to maintain the breeding is described by Greene *et al*.^17^ The diet consists of ingredients such as white beans, wheat germ, and soy protein. The adults were placed in 200 mm PVC tubes, lined with A4 paper sheets, and covered with voile fabric. For feeding the adults, a nutrient solution composed of beer, honey, sucrose, ascorbic acid, nipagin, and water is used, as described by Greene *et al* ^17^ and provided on a piece of cotton soaked and placed in a Petri dish (100 × 150 mm). The breeding was maintained under controlled environmental conditions: a temperature of 25 ± 1 °C, a relative humidity of 60 ± 10%, and a photoperiod of 14 h of light and 10 h of darkness.

### 2.2 Molecular docking of the GORE 3 and trypsin‐*like* from *Spodoptera frugiperda*

Since no crystallographic structures of *S. frugiperda* trypsins are available in the Protein Data Bank (PDB), four distinct trypsin isoforms from this species (XP_050552352.1, ACR25157.1, QLC28936.1, and XP_050550273.1) were retrieved from NCBI and modeled by homology using the Phyre2 platform. Each model was subjected to stereochemical and structural validation by ProSA‐web (Z‐score, overall energy profile) and Ramachandran plot analysis to ensure conformational quality (Supplementary Material S1).

The synthetic peptide GORE 3 was constructed based on its amino acid sequence, designed to incorporate hydrophobic residues, given the reported preference of insect trypsins for hydrophobic amino acids and basic residues such as lysine and arginine. The peptide structure was built and energy‐minimized using the PyRx preparation module.

Benzamidine, a benchmark competitive inhibitor of trypsin, was included as a positive control. Its ligand file was prepared with Open Babel using the same pipeline applied to GORE 3 to ensure a fair comparison: starting from the PubChem SMILES, we generated a 3D conformer, set the dominant amidinium protonation state at the assay pH, performed a short MMFF94 geometry optimization, assigned Gasteiger partial charges, and exported the structure for docking (PDBQT). Docking was then performed against the same receptor, grid definition, and search parameters used for GORE 3, so any differences in pose or score can be attributed to chemistry rather than preprocessing artifacts.

AutoDock Vina was used via PyRx (rigid receptor/flexible ligand). For each isoform, the search box was centered on the catalytic His‐Asp‐Ser triad and sized to cover S1/S1′ and adjacent subsites, accommodating the peptide. Parameters were set to num_modes = 20 and energy_range = 3 kcal/mol; at least three independent runs with different random seeds were performed while keeping the same grid and search settings. The poses from all runs were pooled and clustered using a 2.0 Å heavy‐atom RMSD cutoff. For each cluster, we recorded the population and the median Vina score; the centroid (the pose with the lowest average RMSD to its members) was taken as the representative. The pose used for the interaction diagrams was selected by a multi‐criteria rule: we chose the RMSD = 0.00 Å pose (centroid of the most populated cluster) when its energy was within 0.5 kcal mol^−1^ of the global minimum and it was reproducible across runs; when a less‐populated cluster scored ≥1.0 kcal mol^−1^ better while preserving canonical S1/S1′ recognition, that centroid was selected and its population and energy difference were reported.

Interaction analyses of the docked complexes were conducted in Discovery Studio Visualizer 2017 (Biovia, San Diego, CA, USA – academic license). Hydrogen bonds, hydrophobic contacts (π–π stacking, π–alkyl, alkyl), and π–sulfur interactions with catalytic and substrate‐binding residues were identified. This was performed for both GORE 3 and benzamidine against all four modeled trypsins, allowing a comparative evaluation of binding modes and reproducibility across isoforms.

### 2.3 *In vitro* analyses of the inhibitory effect of the GORE 3

#### 2.3.1 Partial purification of trypsin‐*like* from *Spodoptera frugiperda*

A total of 75 fifth‐instar caterpillars of *S. frugiperda* were dissected, and their intestines were removed. For the preparation of the crude extract, the intestines were macerated in liquid nitrogen. After maceration, they were placed in a 1 mM HCl solution at pH 3, with a ratio of five intestines to solution (1 mL). Immediately thereafter, the samples were centrifuged at 10 000 × *g* for 30 min at 4 °C and subsequently filtered through a 0.22 μm syringe filter. The supernatant was used for the analysis. The partial purification process was carried out by subjecting the enzymatic extract to affinity chromatography on a Benzamidine Sepharose 4 Fast Flow (High Sub) column (2 mL) from Cyntiva (Marlborough, MA, USA), equilibrated with 0.05 M Tris–HCl buffer, pH 7.4, containing 0.5 M NaCl. The proteins were eluted in a continuous flow of 0.05 M glycine buffer, pH 3.0. The fractions corresponding to the activity peaks were concentrated and used in the enzymatic kinetic assays.

The protein concentrations of the crude extracts from different treatments were measured using the bicinchoninic acid (BCA) method, with a standard curve prepared from bovine serum albumin (BSA) (0.2 mg mL^−1^), covering concentrations ranging from 0 to 1000 μg mL^−1^, according to the manufacturer's guidelines (Sigma‐Aldrich, St. Louis, MO, USA).

The readings were performed on a spectrophotometer at a wavelength of 562 nm. Next, samples of the enriched intestinal enzyme extract were run through a one‐dimensional gel made of 12.5% polyacrylamide with SDS (0.1%). The gel was stained with Coomassie Blue solution.

#### 2.3.2 Enzymatic kinetics and inhibition model

Enzymatic activities were observed using the chromogenic substrate DL‐BApNA. The tests were conducted in a reaction medium containing 0.1 mol L^−1^ Tris–HCl, 20 mmol L^−1^ CaCl₂, pH 8.2, and *DL*‐BA*p*NA at concentrations of 0.1, 0.2, 0.4, 0.6, 0.8, and 1.0 μM. To determine the optimal amounts of GORE 3 for testing, a trial was conducted to identify the most suitable concentration range. The concentrations of the inhibitors were GORE 3 at 150, 200, 300, 400, and 500 (μM) and benzamidine at 10, 20, 30, 40, and 50 (μM). At 25 °C, we performed the absorbance reading at 410 nm for 150 s. The activity was measured by the amount of p‐nitroanilide product formed, using a specific value of 8800 M^−1^ cm^−1^.^18^ The specific activity was determined by dividing the absorbance values by the total protein concentration.^19^ The assays were performed in triplicate, including reactions without the addition of inhibitors as a control.

The inhibition models were determined using Michaelis–Menten plots, Lineweaver‐Burk double reciprocal plots, and Dixon plots. The observed enzymatic reaction rates *versus* substrate concentration data (in the presence and absence of inhibitors) were simultaneously fitted using non‐linear regression in OriginPro 2025 software (OriginLab Corporation, Northampton, MA, USA ‐ used under an academic institutional license).

#### 2.3.3 Determination of the inhibition concentration (IC_50_ )

The inhibitory concentration (IC₅₀) was determined by measuring the enzyme's activity with varying amounts of GORE 3. To determine this, we assessed the enzyme's activity by measuring its activity in specific units (U mg^−1^ of protein) with varying amounts of the inhibitor, while maintaining all other conditions constant. The obtained values were normalized relative to the control (assay without inhibitor), which was considered 100% activity. The tests used the substrate *DL*‐BA*p*NA at a final concentration of 1.0 mM and different amounts of the GORE 3, specifically: 150, 200, 300, 400, 500, 600, 700, 800, and 900 (μM). The relative activity was determined for each test point, and a graph was then created to show how enzyme activity changed with varying amounts of the inhibitor, with the inhibitor amount on the x‐axis and the enzyme activity on the y‐axis. The resulting curve was fitted by nonlinear regression using a four‐parameter logistic equation, with the (IC₅₀) value determined as the concentration of the inhibitor responsible for reducing enzyme activity by 50%. All assays were performed in triplicate, and the data were analyzed using ANOVA followed by a mean comparison using the Dunnett test. Calculation of the IC₅₀ was performed using Origin 2025 software.

### 2.4 *In vivo* analyses of the inhibitory effect of GORE 3

#### 2.4.1 Determination of proteolytic activity in midgut extracts after exposure to the inhibitor

For each treatment, 10 insects were kept feeding on artificial diets prepared containing 0.30% (w/v) of the GORE 3 inhibitor and benzamidine. The midguts were dissected when the larvae reached the fifth pupal instar and were grouped in pairs to assess proteolytic activity (total proteases), with a total of five repetitions. The midguts were mechanically crushed with the aid of a pestle and liquid nitrogen. For each sample (i.e., two midguts, one repetition), distilled water (500 μL) at pH 3 was added, and the mixture was centrifuged for 30 min at 10 000 × *g*. The supernatant was collected for further analysis.

Total proteolytic activity was measured using Azocasein at a final concentration of 0.5% in a buffer solution with a pH of 8.2, which also had 20 mM CaCl2, and was kept at 37 °C. The reaction mixture consisted of 450 μL of the substrate and the enzymatic extract (300 μL). After 30 min of incubation, the reaction was stopped by the addition of 10% (w/p) trichloroacetic acid (1000 μL). Next, the samples were vortexed and centrifuged at 16 000 × *g* for 15 min at 25 °C to remove the precipitate. An aliquot of the supernatant (1000 μL) was transferred to microtubes containing 0.5 M NaOH (1000 μL). The absorbance was measured at a wavelength of 440 nm. All measured proteolytic activities were divided by the absorbance of the total protein content of the samples to obtain specific activities. The total protein was measured using bovine serum albumin (BSA) to create a standard curve.^19^

#### 2.4.2 Bioassay of inhibition of trypsin‐*like* proteases

To evaluate the inhibition of trypsin‐*like* proteases in the intestines of *S. frugiperda* larvae at the different concentrations mentioned in item (Section 2.3.2), six fifth‐instar caterpillars were dissected for each treatment, with every two intestines considered a biological replicate, upon reaching the fifth instar. The enzymatic extract was obtained from the dissected intestines of the caterpillars, which were frozen and macerated with liquid nitrogen. The samples were placed in a 1 mM HCl solution at pH 3 at 4 °C (maintaining the ratio of five intestines per 1 mL of solution). Immediately after, the samples were centrifuged at 10 000 × *g* for 30 min at 4 °C and filtered through a 13 mm membrane. The pellets were discarded, and the supernatants containing the enzymatic extracts were separated and stored for further evaluation. To calculate the Michaelis–Menten constant (K_M_), a 0.1 M Tris–HCl buffer solution, pH 8.2, containing 20 mM CaCl₂ was used. The chromogenic substrate *DL*‐BA*p*NA at concentrations of 0.1, 0.2, 0.4, 0.8, and 1.0 mM and the obtained enzymatic extract (10 μL). The measurements were taken three times at 410 nm for 150 s, using a specific value of 8800 M^−1^ cm^−1^, as reported in the work of Almeida^5^ and Meriño‐Cabrera.^20^

#### 2.4.3 Survival analysis

The survival analysis of the caterpillars was conducted based on the number of individuals alive over time, throughout the entire cycle of *S. frugiperda*. Fifteen specimens were used for each treatment, totaling 195 caterpillars, which were observed daily and recorded for any death events. The treatments were divided into six concentrations for GORE 3: 0.002417, 0.01218, 0.02436, 0.04873, 0.1216, and 0.2432% (w/v); six concentrations for benzamidine: 0.0000135, 0.000067, 0.00013, 0.00026, 0.00067, and 0.00134% (w/v); and the control (water).

The survival data were organized into spreadsheets containing the number of individuals alive each day for each treatment. Based on these data, survival curves were constructed using the Kaplan–Meier method in the software OriginPro 2025. The curves represented the proportion of surviving individuals over time for each experimental group. For the statistical comparison between the treatments, the log‐rank test (Mantel‐Cox) was applied, with a significance level of 5% (*P* < 0.05), to identify significant differences in the survival patterns. The obtained curves were graphically represented, allowing the visualization of the differences in mortality rates between the evaluated treatments.

#### 2.4.4 Biological cycle and body mass of caterpillars and pupae

The duration of the larval period was determined through daily observations. The period began with the egg hatching and ended with the formation of the pupa, as indicated by body retraction and the initiation of pupal integument sclerotization. The data were recorded individually, counting the total number of days between hatching and pupation. The newly formed pupae were transferred to clean and labeled containers and kept under the same environmental conditions until the adults emerged. The pupal period was determined as the interval between the pupation date and the emergence of the adult. Each pupa was monitored individually until emergence.

The average weight of the caterpillars was determined after 10 days of feeding on diets containing the different treatments with the inhibitors benzamidine and GORE 3, as well as the control group. The weight values (in grams) were used to calculate the mean and standard deviation of each experimental group. The weight of the pupae was evaluated as a development parameter after the larval stage.

The surviving caterpillars from each treatment were kept individually until pupation and then weighed on an analytical balance 24 h after the formation of the pupa. The weight data (in grams) were recorded, and subsequently, the mean and standard deviation of the pupal weight for each experimental group were calculated. The results were represented by bar charts constructed using the OriginPro 2025 software. The differences between the groups were checked using the Kruskal–Wallis test and Dunn's multiple comparisons test, with a significance level of 5% (*P* < 0.05). Groups with significant differences were identified by different letters above the bars in the graph.

#### 2.4.5 Nutritional parameters

The analysis of the caterpillars' nutritional parameters was based on food consumption and body mass gain over a specified period. For this, the following data were considered: the initial weight of the caterpillars, the final weight after 24 h of feeding, the weight of the provided diet, the weight of the remaining diet, and the weight of the feces. These data were subjected to the following calculations: efficiency of conversion of ingested food —ECI = (B/I) × 100; efficiency of conversion of digested food —ECD = (B/I – F) x 100; and approximate digestibility —AD = (I‐F/I) × 100.

For the trial, 15 specimens were used per treatment, totaling 105 caterpillars. The following concentrations were evaluated: 0.10, 0.20, and 0.30% (w/v) for GORE 3 and benzamidine. The caterpillars were individually placed in plastic trays containing 16 wells as soon as they hatched and were fed the artificial diet described in previous sections until they reached the third larval instar. Soon after, the diets were replaced with those containing the incorporated inhibitor concentrations.

The values obtained for each parameter were tabulated and subjected to statistical analysis to determine whether there were significant differences between the treatments. The statistical analyses were performed using OriginPro 2025 software, which applied the non‐parametric Kruskal–Wallis test followed by the Dunnett multiple comparisons test, with a significance level of 5% (*P* < 0.05).

#### 2.4.6 Consumed leaf area

For the execution of this trial, 30 specimens were used for each treatment, totaling 90 caterpillars. The number of leaves eaten was measured using a solution that had a 0.30% (w/v) concentration of GORE 3 and benzamidine, plus a control that was just water with 0.01% Tween 80®.

The caterpillars were placed individually in plastic trays containing 16 wells, and once hatched, were fed the artificial diet described in the previous sections until they reached the third larval instar. Before the trial, the caterpillars were starved for 24 h, and after being placed on the leaf discs, they were left for 4 h before the results were evaluated. The corn leaves in the vegetative V8 phase were donated by the Insect‐Microorganism Interactions laboratory located at the Federal University of Viçosa.

The dependent variable, leaf area consumed, was analyzed using mixed models to investigate the effects of the experimental treatments: control, benzamidine, and GORE 3. To measure the leaf area consumed, ImageJ was used before and after the leaves were analyzed. Initially, the normality of the residuals was assessed using the Shapiro–Wilk test. A mixed linear model (LMM) was fitted using the Lmer() function from the Ime4 package. Finally, we applied the Kruskal–Wallis test as a non‐parametric approach to verify whether there were significant differences between the treatments.

Additionally, the pair preference index for each treatment was calculated using the formula PI = 2 T/(T + C), where T and C represent the tested treatment and the control, as described by Bortoli.^21^ Additionally, the variation coefficients (CV) for the pairs being tested and the most minor significant difference (MSD) at a 5% significance level were calculated to assess differences between the treatments.

## 3 RESULTS

### 3.1 Molecular docking of the GORE 3 and trypsin‐*like* from *Spodoptera frugiperda*

**Figure 1:**
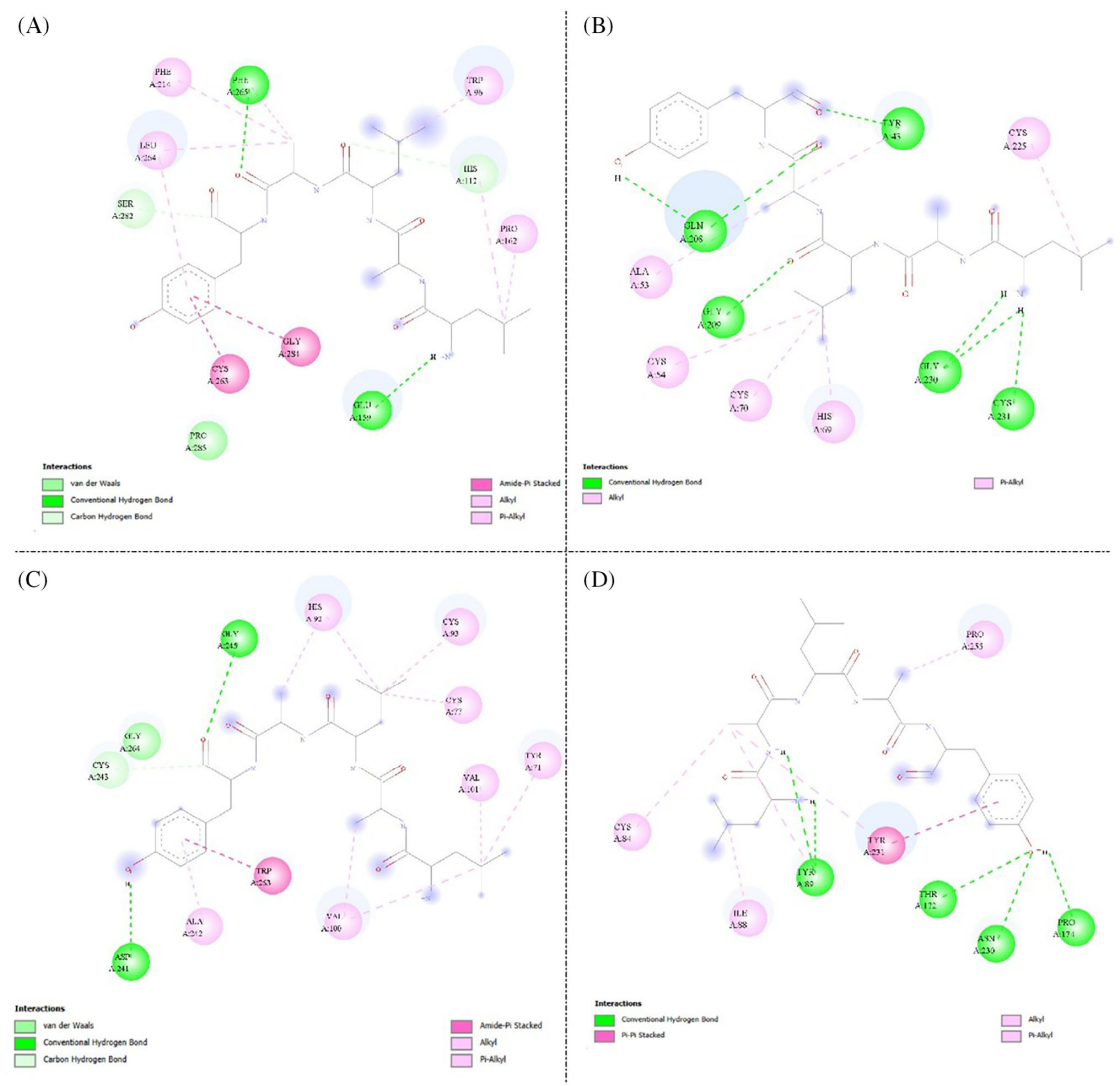
Two‐dimensional interaction diagrams of the GORE 3 peptide docked against four modeled trypsin isoforms from Spodoptera frugiperda. (A) Isoform XP_050552352.1, (B) Isoform ACR25157.1, (C) Isoform QLC28936.1, (D) Isoform XP_050550273.1. Hydrogen bonds are shown in green, hydrophobic interactions (alkyl, π–alkyl, π–π stacked) in pink/purple, carbon hydrogen bonds in light green, and π–sulfur interactions in orange. The diagrams highlight the conserved binding of GORE 3 within the catalytic cleft of the proteases.

The two‐dimensional diagrams in (Fig. 1) show that the GORE 3 peptide conservatively recognizes the catalytic cleft of the four modeled trypsins from S. frugiperda (XP_050552352.1, ACR25157.1, QLC28936.1, and XP_050550273.1). Across all isoforms, a well‐defined network of hydrogen bonds (green) with polar residues lining the S1/S1′ pocket is accompanied by numerous hydrophobic contacts—alkyl, π–alkyl, and amide–π/π–π stacking (magenta/violet)—distributed along the groove walls. Complementary carbon–hydrogen bonds (light green) and, depending on the isoform, π–sulfur interactions with nearby cysteines (orange) further contribute to ligand anchoring. Although subtle differences are evident among panels, the overall pattern is consistent: GORE 3 occupies the catalytic channel with an extensive interaction mesh that stabilizes its orientation within the active site.

**Figure 2:**
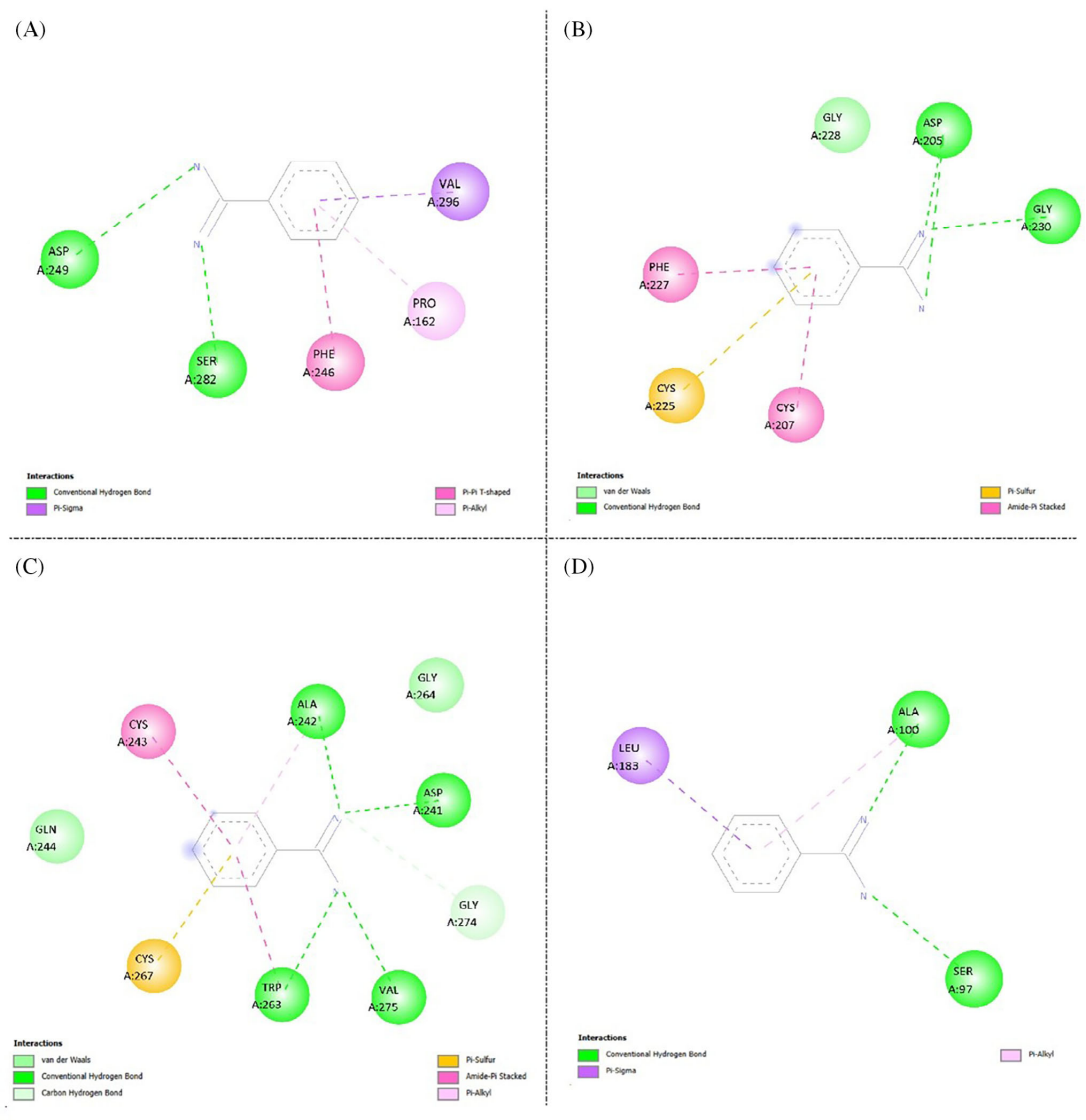
Two‐dimensional interaction diagrams of benzamidine docked against four modeled trypsin isoforms from Spodoptera frugiperda. (A) Isoform XP_050552352.1, (B) Isoform ACR25157.1, (C) Isoform QLC28936.1, (D) Isoform XP_050550273.1. Conventional hydrogen bonds are represented in green, carbon hydrogen bonds in light green, hydrophobic contacts (π–alkyl, alkyl, π–π T‐shaped) in pink/purple, π–sulfur interactions in orange, and amide–π stacked interactions in violet.

By contrast, (Fig. 2) shows that benzamidine, used as a control, reproduces the canonical trypsin‐recognition mode with fewer total contacts. One to a few directed hydrogen bonds (green) to the S1 pocket predominate, together with a moderate hydrophobic wrap involving π–alkyl, alkyl, and T‐shaped π–π contacts (magenta/violet); in some complexes, carbon–hydrogen bonds (light green), π–sulfur interactions (orange), and amide–π stacking (violet) are also observed. Taken together, the diagrams suggest that although both ligands fit the same pocket, GORE 3 establishes a denser and more diverse interaction network than benzamidine.

These structural findings agree with the docking affinities in Table 1 nd. GORE 3 yielded binding energies between −9.1 and − 7.9 kcal mol^−1^ across the four isoforms (mean − 8.25 ± 0.57 kcal mol^−1^), whereas benzamidine ranged from −6.1 to −5.2 kcal mol^−1^ (mean − 5.45 ± 0.44 kcal mol^−1^). The advantage of GORE 3 was consistent across proteases, with an average difference of −2.8 ± 0.18 kcal mol^−1^ relative to the control. Assuming ΔG ≈ RT ln Kd, this gap translates into an approximate two‐order‐of‐magnitude improvement in affinity. For both ligands, the best score was observed for isoform 1. Overall, the combination of a richer interaction network and more favorable docking energies supports that GORE 3 binds more stably and uniformly than benzamidine to *S. frugiperda* trypsins.

**Table 1:** Comparison of affinity energy (ΔG, kcal mol^−1^) of GORE 3 *vs.* benzamidine across *S. frugiperda* trypsin isoforms

Complex Enzyme‐ligand	Affinity energy (Kcal mol^−1^)
**Ligand: GORE 3**	
*S. frugiperda 1*	−9.1
*S. frugiperda 2*	−8.1
*S. frugiperda 3*	−7.9
*S. frugiperda 4*	−7.9
**Ligand: Benzamidine**	
*S. frugiperda 1*	−6.1
*S. frugiperda 2*	−5.2
*S. frugiperda 3*	−5.3
*S. frugiperda 4*	−5.2

#### 3.1.1 Partial purification of trypsin‐*like* from *Spodoptera frugiperda*

The partial purification of trypsin‐*like* enzymes from the crude extract of the midgut of *S. frugiperda* resulted in a significant increase in specific activity and the degree of purification. The affinity chromatography on benzamidine sepharose increased the total activity from 4.32 × 10^−8^ to 3.37 × 10^−8^, while also significantly reducing the total amount of proteins from 153.05 mg to 14.90 mg, which means the active components became more concentrated. As a result, the specific activity increased from 2.82 × 10^−10^ U mg^−1^ to 2.26 × 10^−9^ U mg^−1^, representing an approximate 8.03‐fold gain in the degree of purification compared to the initial crude extract and a calculated yield of 78.12%, as described in Table 2.

**Table 2:** Partial purification of the crude extract from the intestine of *S. frugiperda*

Purification step	Total activity (U)	Total protein (mg)	Yield (%)	Specific activity (U/mg)	Purification fold
Crude extract (filtered)	4.32 × 10^−8^	153.0477	100	2.82 × 10^−10^	1
Benzamidine Sepharose	3.37 × 10^−8^	14.8989	78.12	2.26 × 10^−9^	8.03

The obtention of trypsin‐*like*, using the HiTrap Benzamidine Sepharose affinity column, through protein quantification and enzymatic activity reading in the crude extract and the purified fraction.

**Figure 3:**
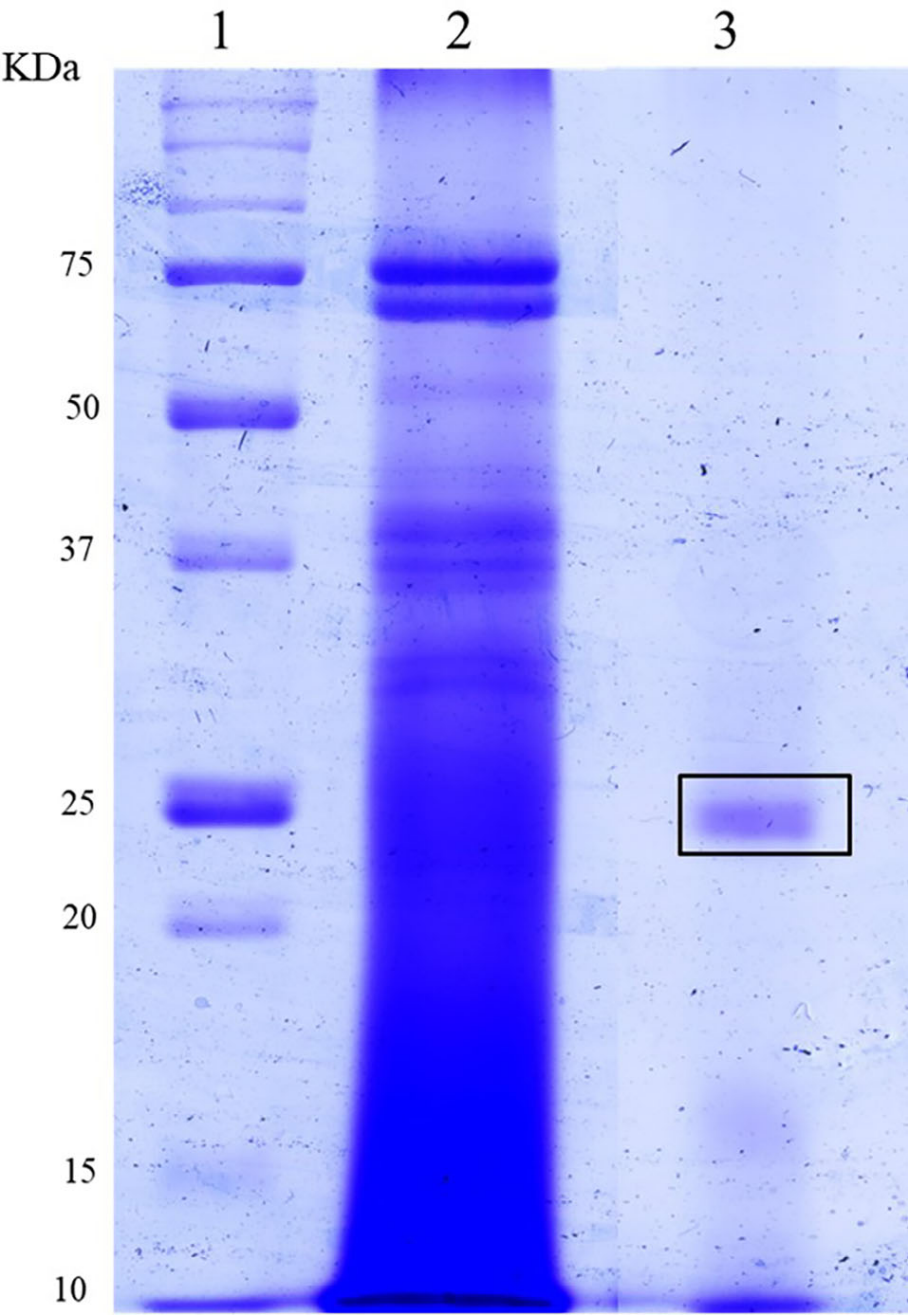
Electrophoretic profile in sodium dodecyl sulfate‐polyacrylamide gel electrophoresis (SDS‐PAGE). Electrophoretic profile of the intestine of *Spodoptera frugiperda* (Lepidoptera: Noctuidae) on HiTrap Benzamidine column (high sub) (GE). (1). Marker (2). Crude extract (3). purification on HiTrap Benzamidine column (high sub).

Next, after passing through the chromatography column, we collected the enriched fractions containing trypsin‐*like* activity and evaluated them using SDS‐PAGE. Lane 2 of the gel, containing the purified extract, exhibited a distinct protein profile compared to lane 3, which included the crude extract. In the purified extract, we observed more distinct bands between 20 and 35 kDa than in the crude extract, suggesting that trypsin‐*like* enzymes are present and that fewer other proteins are present (Fig. 3).

### 3.2 Enzymatic kinetics and inhibition model

**Figure 4:**
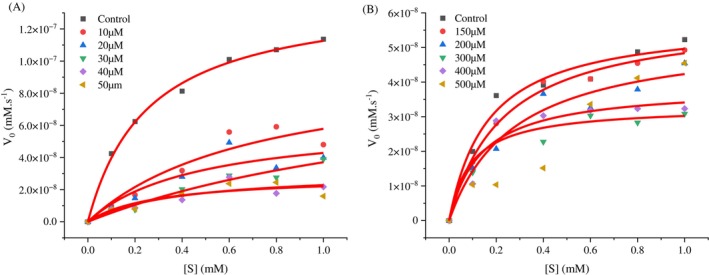
Michaelis–Menten curve for substrate concentrations. Substrate concentrations of *DL*‐BA*p*NA ranging from 0.1‐1 mM, GORE 3 from 10 – 50 μM (A) and 150 – 500 μM (B). (A) Benzamidine, (B) GORE 3.

**Figure 5:**
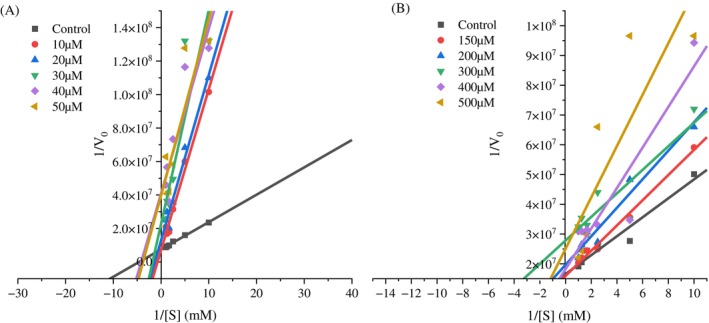
Linear representations of the kinetic data through the Lineweaver‐Burk plot (1/V₀ *vs*. 1/[S]). Linear representation for substrate concentrations of *DL*‐BA*p*NA ranging from 0.1‐1 mM, GORE 3 from 10 – 50 μM (A) and 150 – 500 μM (B). (A) Benzamidine, (B) GORE 3.

**Figure 6:**
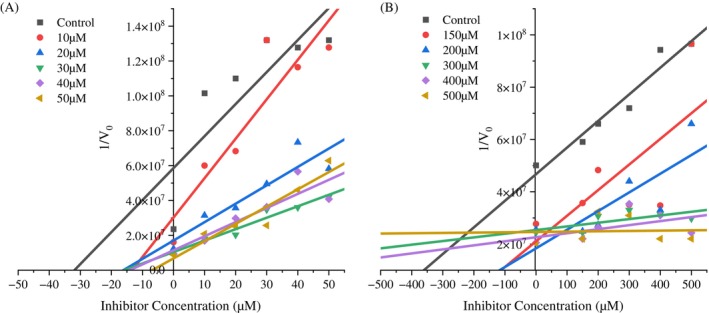
Dixon plots (1/V₀ *versus* inhibitor concentration). Obtained for the characterization of the type of inhibition for substrate concentrations of *DL*‐BA*p*NA ranging from 0.1‐1 mM and the inhibitor from 10 – 50 μM (A) and 150 – 500 μM (B). (A) Benzamidine, (B) GORE 3.

A non‐linear regression method was employed to assess the inhibition kinetics of the peptides against trypsin‐*like* enzymes from *S. frugiperda* and to determine the inhibition constant (K_i_). We analyzed the control data (without inhibitor) using nonlinear regression to obtain the best fit values for Vmáx_(app)_ and K_M(app)_ (Fig. 4(A), (B)) and (Fig. 5(A), (B)). These values were then fixed in the subsequent analysis of all data (control and inhibition data) for the calculation of K_i_. The K_i_ value was 16.49 μM for the inhibitor benzamidine (Fig. 6(A)) and 4.0 mM for the inhibitor GORE 3 (Fig. 6(B)). The regression analysis indicates that both peptides function as competitive inhibitors for *S. frugiperda* trypsin, as demonstrated by the kinetic enzyme analysis using OriginPro 2025 software.

#### 3.2.1 Inhibition concentration (IC_50_ )

To evaluate the inhibitory potential of the peptide GORE 3 on the activity of trypsin‐*like* intestinal proteases of *S. frugiperda*, partially purified trypsin fractions, *DL*‐BA*p*NA substrate, and different concentrations of benzamidine (0.2, 0.4, 0.6, 0.8, 10, 20, 30, 40, 50, and 60 μM) and GORE 3 (150, 200, 300, 400, 500, 600, 700, 800, and 900 μM) were used.

**Figure 7:**
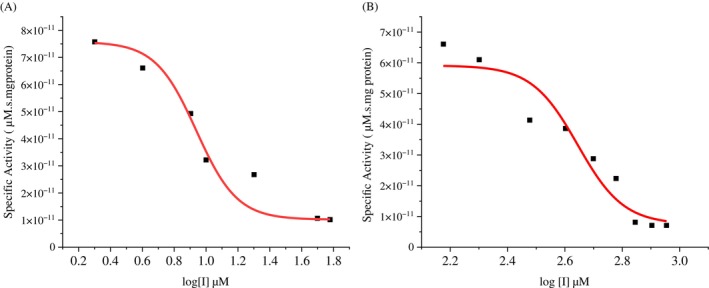
Enzymatic inhibition curve of the inhibitor benzamidine and GORE 3. The specific activity of the enzyme was plotted as a function of the inhibitor concentration log [I] (μM). The curve was fitted using the sigmoidal dose–response model (Hill Equation). The IC₅₀ value was determined to be 8.58 μM for benzamidine and 433.98 μM for GORE 3. The points represent the experimental values, and the red line corresponds to the nonlinear fit. The coefficient of determination R^2^ = 0.97571.

The results showed that GORE 3 is less effective at inhibiting enzymes than benzamidine, which reduces enzyme activity by 50% at a concentration of 8.58 μM (Fig. 7(A)). GORE 3 was able to significantly reduce trypsin activity at higher amounts, making it a potential inhibitor for pest control methods. Benzamidine inhibited *S. frugiperda* protease activity by 89% at 60 μM, whereas GORE 3 required 700 μM to achieve a comparable level of inhibition. The IC_50_ value for the GORE 3 peptide was 433.98 μM (Fig. 7(B)).

### 3.3 Proteolytic activity in midgut extracts after exposure to the inhibitor

**Figure 8:**
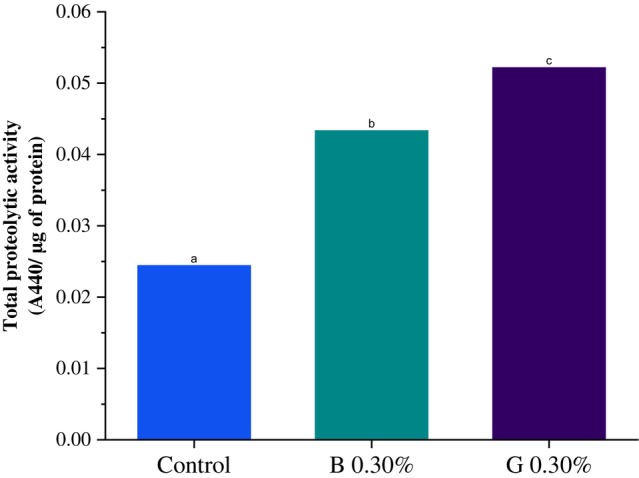
The total protease activities of midgut extracts from *Spodoptera frugiperda*. Whether exposed to IPs GORE 3 (G) and benzamidine (B) or not, were measured at a concentration of 0.30% (w/v). The values are averages. Different letters indicate differences between the treatments. The differences between the groups were analyzed using the Tukey test. Different letters indicate a statistically significant difference between treatments (*P* < 0.05).

The activity of total proteases was significantly increased in *S. frugiperda* caterpillars after 20 days of feeding on an artificial diet containing the inhibitors GORE 3 and benzamidine at a concentration of 0.30% (w/v) (Fig. 8).

### 3.4 Bioassay of trypsin‐*like* protease inhibition

**Figure 9:**
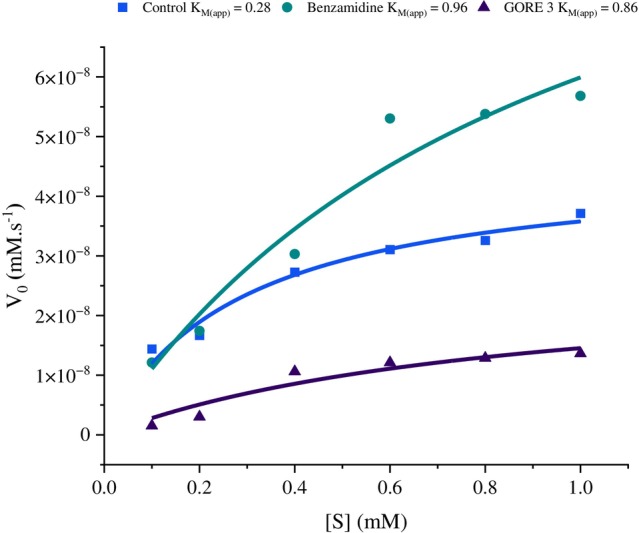
Michaelis–Menten plot analyzes the affinity of trypsin‐like proteases extracted. Intestines from *Spodoptera frugiperda* using *DL*‐BA*p*NA as a substrate, following exposure to the inhibitors GORE 3 and benzamidine, which were added to the artificial diet at a concentration of 0.30% (w/v).

The tests showed that trypsin‐*like* proteases from *S. frugiperda* had a low affinity to the substrate *DL*‐BA*p*NA when caterpillars were fed an artificial diet with 0.30% of the inhibitors benzamidine and GORE 3. The control group had a K_M(app)_ of 0.28 mM, while benzamidine had an average K_M(app)_ of 0.96 mM, and GORE 3 had an average K_M(app)_ of 0.86 mM. The control group had the K_M(app)_ = 0.28 mM, while benzamidine had an average K_M(app)_ = 0.96 mM and GORE 3 had an average K_M(app)_ = 0.86 mM (Fig. 9).

### 3.5 Survival curve

The survival analysis, conducted using the Kaplan–Meier estimator, indicates significant differences in the survival percentage of GORE 3 compared to the control. The concentrations 0.00241% (log‐rank χ^2^ = 5.42, *P* = 0.019) and 0.04873% (log‐rank χ^2^ = 4.27, *P* = 0.038) resulted in a mortality rate of approximately 47%. The concentration of benzamidine that showed significant mortality was 0.000067% (log‐rank χ^2^ = 5.41, *P* = 0.019), resulting in a mortality percentage of 0.000067%.

**Figure 10:**
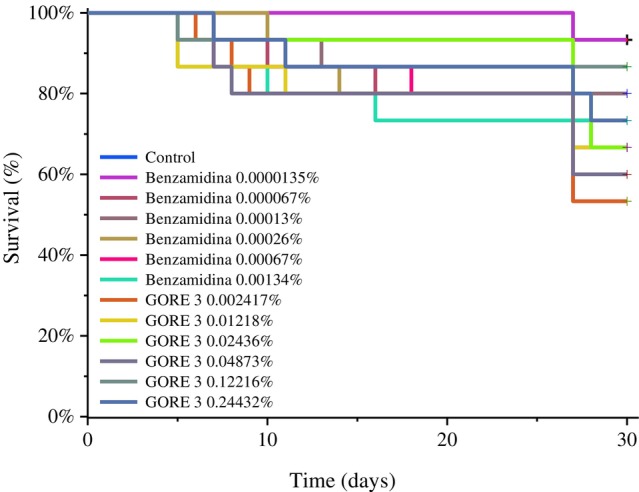
Survival curve of *Spodoptera frugiperda* (Lepidoptera: Noctuidae). After exposure to the inhibitors GORE 3 and benzamidine at various concentrations: 0.000013%, 0.000067%, 0.00013%, 0.00026%, 0.00067%, and 0.00134% (w/v) for benzamidine, and 0.00241%, 0.01218%, 0.02436%, 0.04873%, 0.12216%, and 0.24432% (w/v) for GORE 3.

There was also a significant difference between the results of benzamidine at 0.000067% compared to benzamidine at 0.00013% (log‐rank χ^2^ = 6.18, *P* = 0.012); GORE 3 at 0.00241% compared to benzamidine at 0.00013% (log‐rank χ^2^ = 6.18, *P* = 0.012); and GORE 3 at 0.04873% compared to benzamidine at 0.00013% (log‐rank χ^2^ = 4.74, *P* = 0.029. The treatments that showed low mortality rates were the higher concentrations of GORE 3 at 0.1216% and 0.2432% (w/v), which resulted in 6.7% and 26% mortality, respectively (Fig. 10).

### 3.6 Biological cycle and body mass of caterpillars and pupae

**Figure 11:**
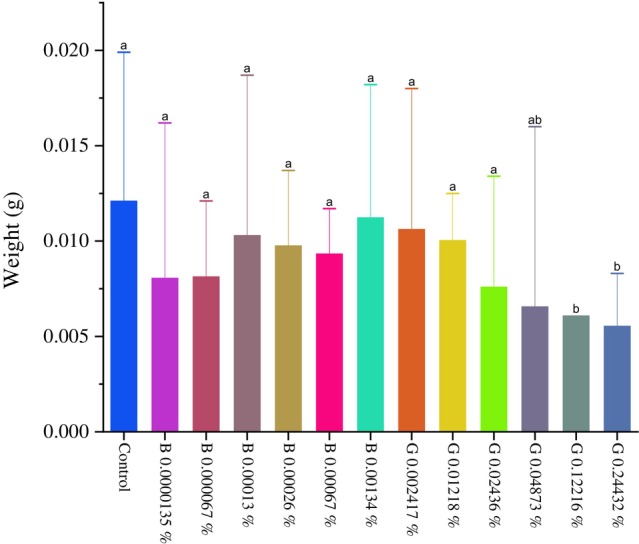
Graph of larval mass (g) of *Spodoptera frugiperda*. Fed an artificial diet containing GORE 3 (G) and benzamidine (B) at concentrations of 0.000013%, 0.000067%, 0.00013%, 0.00026%, 0.00067%, and 0.00134% (w/v) for benzamidine and 0.00241%, 0.01218%, 0.02436%, 0.04873%, 0.12216%, and 0.24432% (w/v) for GORE 3. The differences between the groups were analyzed using the Dunn test with correction for multiple comparisons. Different letters indicate a statistically significant difference between the treatments (*P* < 0.05).

Significant differences (*P* < 0.0001) occurred in the body mass of fifth instar caterpillars exposed to the peptide GORE 3 at concentrations of 0.04873%, 0.12216%, and 0.24432% (w/v) (Fig. 11). The concentrations of benzamidine did not produce statistical differences compared to the control, indicating that GORE 3 negatively affected the body mass gain of the caterpillars at higher concentrations.

**Figure 12:**
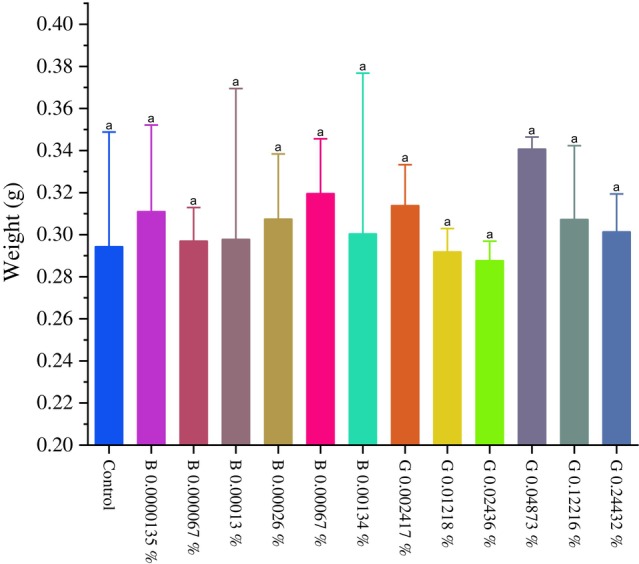
Graph of pupa mass (g) of Spodoptera frugiperda caterpillars. Exposed to GORE 3 and benzamidine inhibitors. Averages of the weight of *S. frugiperda* pupae. The differences between the groups were analyzed using the Dunn test with correction for multiple comparisons. Different letters indicate a statistically significant difference between the treatments (*P* < 0.05).

**Figure 13:**
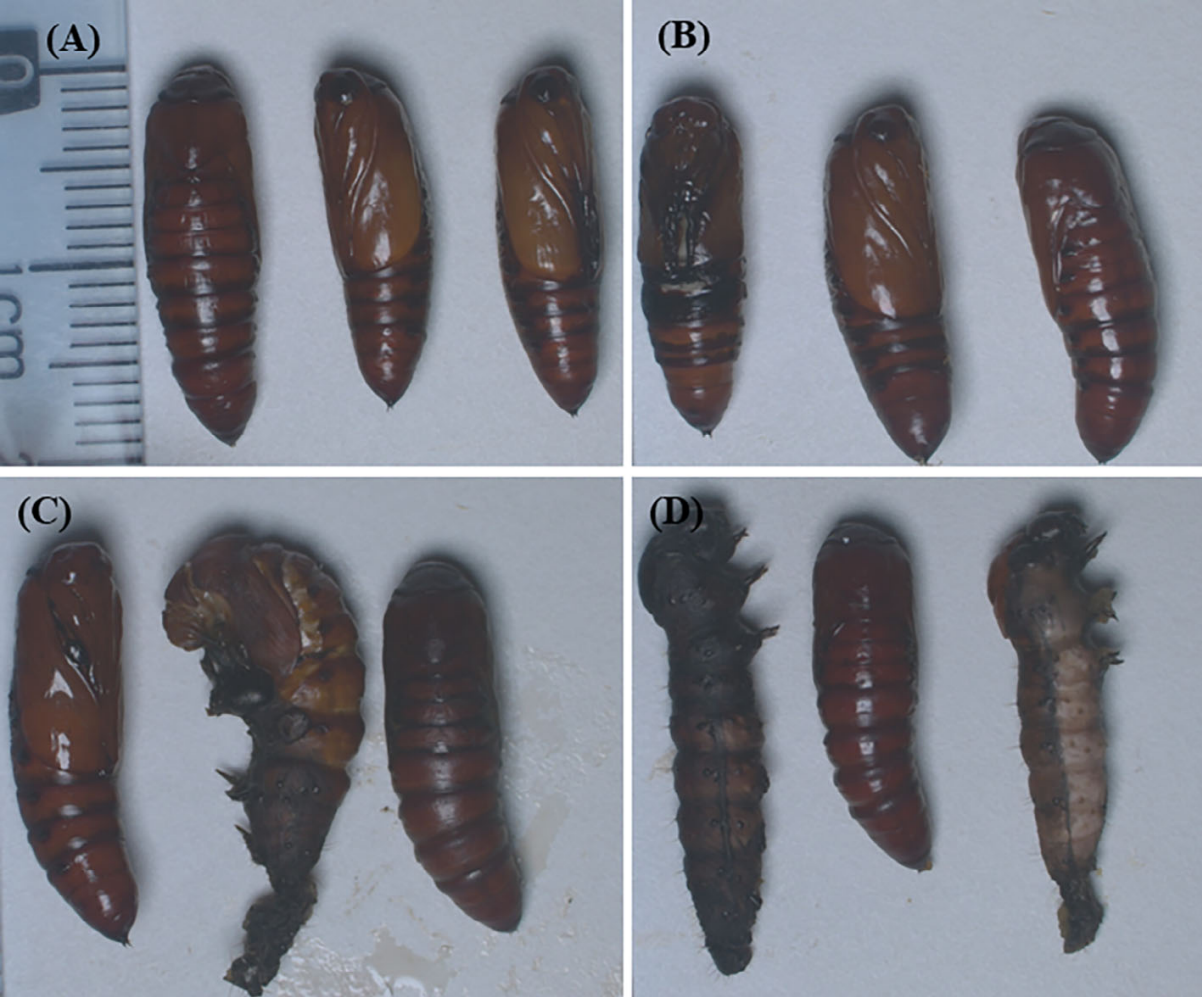
Records of Spodoptera frugiperda pupae. Records taken with the Zeiss stereoscopic using the AxioCam Mrc camera ‐ (A) control, (B) Benzamidina (C) GORE 3 0.02436% (D) GORE 3 0.04873% (w/v).

Regarding the pupa mass, the GORE 3 and benzamidine treatments did not show significant differences (*P* > 0.0001) at any of the tested concentrations (Fig. 12). On the other hand, there were malformations of pupae in the caterpillars that were fed with the GORE 3 inhibitor at concentrations of 0.02436% and 0.04873% (w/v), as recorded in Fig. 13(A)–(D).

**Figure 14:**
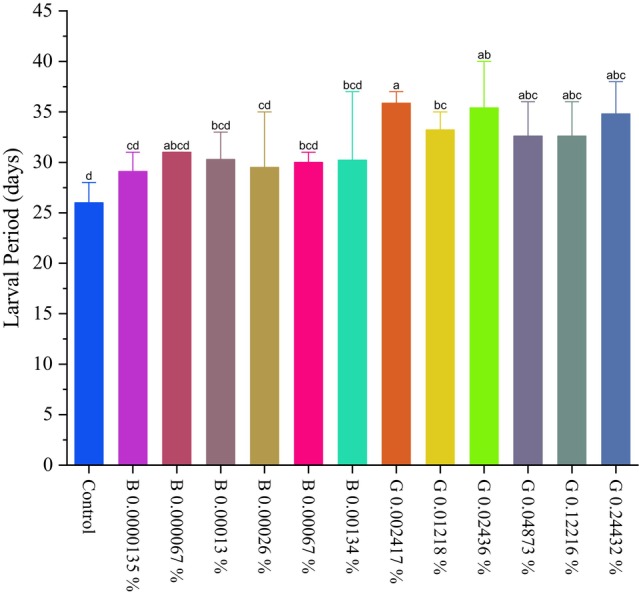
Graph of the larval period of *Spodoptera frugiperda* (Lepidoptera: Noctuidae). Exposed to GORE 3 (G) and benzamidine (B) inhibitors at concentrations of 0.000013%, 0.000067%, 0.00013%, 0.00026%, 0.00067%, and 0.00134% (w/v) for benzamidine and 0.00241%, 0.01218%, 0.02436%, 0.04873%, 0.12216%, and 0.24432% (w/v) for GORE3. The differences between the groups were analyzed using the Dunn test with correction for multiple comparisons. Different letters indicate a statistically significant difference between the treatments (*P* < 0.05).

Exposure to the GORE 3 inhibitor strongly altered the larval period. A prolongation of the larval period is observed in caterpillars exposed to this inhibitor, compared to the control. In GORE 3, all tested concentrations differed statistically from the control (*P* < 0.05), showing an average larval period of 35 ± 0.15 days, while the control showed an average of 25 ± 0.15 days, demonstrating the effect of this peptide on the elongation of the larval cycle caused by this peptide, as presented in (Fig. 14). The concentrations that showed the most remarkable prolongation in the larval period were 0.02436% (*P* < 0.0001), 0.04873% (*P* = 0.0033), and 0.12216% (*P* = 0.0033) (w/v).

### 3.7 Nutritional parameters

**Figure 15:**
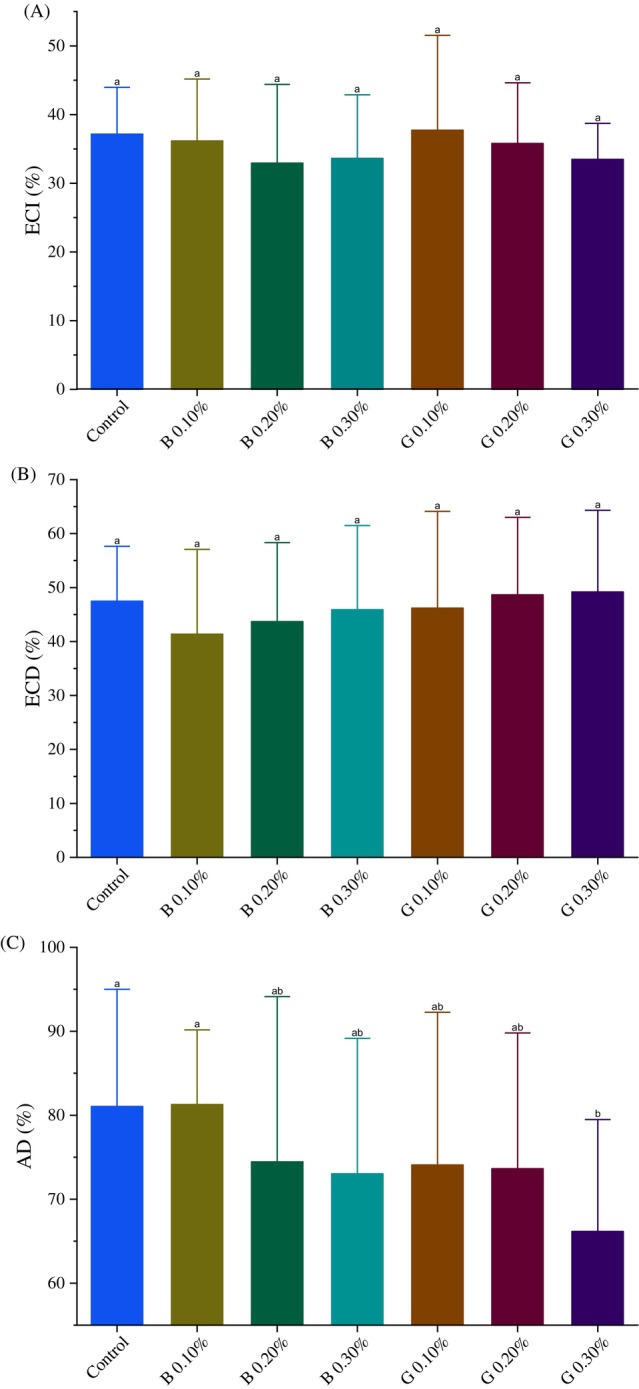
Nutritional indices of Spodoptera frugiperda (Lepidoptera: Noctuidae) larvae. Exposed to a diet containing 0.10%, 0.20%, and 0.30% (w/v) of the peptide GORE 3 (G) and the inhibitor benzamidine (B) and a control diet (without inhibitor). (A) Efficiency of ingested food conversion (EIC); (B) Efficiency of ingested food conversion (ECD); and (C) Approximate Digestibility (AD). The differences between the groups were analyzed using the Dunn test with correction for multiple comparisons. Different letters indicate a statistically significant difference between the treatments (*P* < 0.05).

The tests on nutritional factors to check how well the caterpillars convert the food they eat, and digest showed that the peptide GORE 3 did not harm the caterpillars at concentrations of 0.10, 0.20, and 0.30% (w/v) in the ECI and ECD measurements (*P* > 0.05) (Fig. 15(A), (B)). However, there was a notable change in the approximate digestibility (AD) when the caterpillars were exposed to the 0.30% (w/v) concentration of GORE 3 (Fig. 15(C)). None of the benzamidine inhibitor concentrations showed a significant difference compared to the control group.

### 3.8 Leaf area consumed

The average leaf consumption shows minor variation across treatments, with leaves treated with GORE 3 at 0.30% (w/v) exhibiting the lowest consumption; however, these differences were not statistically significant. Regarding the preference index (PI), which compares the preference between two treatments, benzamidine and GORE 3, there was a higher consumption in the leaves treated with benzamidine (PI = 0.91). When comparing GORE 3 and the control, there was a higher consumption in the control (IP = 0.92). Between benzamidine and the control, there was a higher consumption in benzamidine (IP = 1.02). (Table 3).

**Table 3:** Leaf area (cm^*2*^) consumed and preference indices (PI) by *Spodoptera frugiperda* caterpillars

Comparisons	Leaf area consumed (cm^2^)	IP	DMS (5%)	CV (%)
Benzamidine ‐ Control	1.27 a / 1.23 a	1.02	0.75	98.74
GORE 3 – Control	1.06 a / 1.23 a	0.92	0.71	96.03
GORE 3 ‐ Benzamidine	1.06 a / 1.27 a	0.91	0.74	90.89

The differences between the groups were analyzed using the Dunn test with correction for multiple comparisons. Different letters indicate a statistically significant difference between the treatments (*P* < 0.05).

## 4 DISCUSSION

The fall armyworm *S. frugiperda* poses a global threat to food security, severely impacting food production worldwide.^22^ Although various strategies have been reported in the literature to control this species, it remains a persistent pest and continues to be a worldwide problem.^23^

The search for sustainable, efficient strategies is crucial to developing methods to control this pest. Therefore, studies on protease inhibitors in the intestine of *S. frugiperda* have been conducted to aid in pest management.^24^, ^25^, ^26^, ^27^

This study aimed to evaluate the effectiveness of the peptide GORE 3 in inhibiting the intestinal proteases of *S. frugiperda* through both laboratory and *in vivo* tests. There are examples of peptides that can block these enzymes, remain stable, and kill insects, such as the molecules GORE 1 and GORE 2, as demonstrated in research by Almeida^5^ and Schultz^7^ with the soybean caterpillar *Anticarsia gemmatalis* (Lepidoptera: Noctuidae).

The molecular docking results point to a conserved binding mode of GORE 3 across multiple trypsins from *S. frugiperda*, suggesting robustness against isoform‐specific variability of the target. This convergence is valuable for bioinsecticidal applications: a peptide ligand that maintains recognition across isoforms reduces the risk of escape under selective pressure and can sustain efficacy under the changing biological conditions of the insect midgut (alkaline pH, high ionic strength, presence of cofactors and natural surfactants).^28^ The qualitative comparison with benzamidine further supports that the peptide's greater contact complexity (e.g., engagement of adjacent subsites) translates into additional complex stabilization, a pattern consistent with the literature on peptides engineered against lepidopteran trypsins.

From a rational design standpoint, the observed features guide targeted optimizations: maintain occupancy of S1/S1′ and neighboring subsites; reinforce aromatic and aliphatic contacts that contribute favorable desolvation and stacking; and explore substitutions that preserve geometry and electrostatics while improving proteolytic stability (end‐capping/cyclization, strategic N‐methylations, D‐chiral inversions at non‐critical positions, or i,i + 4 stapling).^29^, ^30^ Such modifications could improve midgut half‐life and local bioavailability without penalizing affinity. Given that different pocket residues determine specificity between insects and mammals, per‐residue energy decomposition would help prioritize *S. frugiperda*–specific hotspots with potential selectivity over trypsins from non‐target organisms.

Nevertheless, inherent limitations of docking must be considered: reliance on the structural models of the isoforms (homology, catalytic‐site protonation at alkaline pH), receptor rigidity during docking, and scoring‐function sensitivity. A natural next step is dynamic and energetic validation: molecular dynamics under midgut‐like conditions (effective pH, 150 mM salts), free‐energy estimates via MM/GBSA or alchemical methods, and time‐averaged contact profiles to verify persistence of key interactions.^31^ In parallel, integrating 3D‐QSAR and peptide pharmacophore inference, as recent studies suggest, can accelerate analogue prioritization.^32^

*In vitro* studies have shown that GORE 3 works by competing with the substrate for the same binding site on the enzyme. The inhibitor and the substrate both attempt to bind to the same site on the enzyme, illustrating how they interact with the enzyme's critical parts.^33^ The values obtained, represented by the inhibition constants, are reflected in the chemical and structural characteristics of each inhibitor. Benzamidine (C₇H₈N₂), a synthetic amidine derivative with the chemical structure, competes with other substrates to block the activity of trypsin‐*like* enzymes. At low concentrations, benzamidine binds to the specific site of trypsin (S1), forming interactions that are both hydrophobic and electrostatic between its amine group and the carboxyl group of Asp 177.^34^ In contrast, GORE 3 is a peptide composed of five hydrophobic amino acids, with the last residue characterized by tyrosine.

Thus, molecular size influences inhibitory capacity, as larger peptides can occupy more binding regions within the enzyme's active site.^31^ In 2022, Almeida Barros conducted studies on docking and molecular dynamics, which demonstrated that the peptides GORE 1 and GORE 2 formed strong connections with the trypsin‐*like* enzymes of *A. gemmatalis*.

Furthermore, studies have shown that peptides containing arginine have a higher inhibitory capacity, reducing the activity of trypsin‐*like* proteases.^31^, ^35^ GORE 3, however, had a much lower inhibition constant than these tripeptides, indicating that the type of amino acids and the size of the peptides influence the inhibition constant values. Therefore, *in vivo* assays are crucial to assess the ability of these peptides to cause both direct and indirect damage in the development of pest insects.

The evaluation of the inhibitory concentration (IC_50_) for the peptide GORE 3 showed an inhibition of 89.85% of the specific activity at the highest tested concentration, while benzamidine exhibited an inhibition of 86.66%. However, it required larger amounts of GORE 3 to reach these high inhibition levels, which supports the findings from the laboratory tests on trypsin‐*like* enzymes and their competitive inhibition model.

*In vivo* tests examined the survival of *S. frugiperda* larvae when fed an artificial diet containing varying amounts of the GORE 3 peptide. Concentrations of 0.00241% and 0.04873% (w/v) influenced survival rates. Consequently, we observed that *S. frugiperda* larvae developed the ability to overexpress proteases as a defense mechanism at higher concentrations.^36^

One of the main concerns regarding the effectiveness of these protease inhibitors against insects is that insects possess numerous protease genes that can produce various types of proteases, which may respond differently to the tested inhibitor.^37^ To determine if more proteases are produced when the inhibitor is at higher levels, a test was conducted to measure the total amount of protease produced by *S. frugiperda* larvae fed an artificial diet containing 0.30% of the GORE 3 inhibitor and benzamidine. To assess whether the larvae produced more proteases when given higher amounts of the inhibitor, a test was performed to measure the total protease produced by *S. frugiperda* larvae consuming an artificial diet with 0.30% of the GORE 3 inhibitor and benzamidine.

The caterpillars fed with 0.30% (w/v) of the inhibitor showed a significant increase in total proteases. These results demonstrate the activation of compensatory mechanisms in the caterpillars in response to the presence of the inhibitors. According to Sultana *et al*.,^38^ herbivorous insects typically respond to the consumption of inhibitors by overexpressing alternative proteases, less sensitive isoforms, and even enzymes that are resistant to inhibitory action. Even so, the concentrations could kill as much as 46.66% of the *S. frugiperda* population at a level of 0.241% (w/v) of the GORE 3 inhibitor, and the same death rate was seen with benzamidine at a level of 0.013% (w/v).

When examining the effectiveness of trypsin‐*like* enzymes with the substrate, *S. frugiperda* larvae that consumed the artificial diet with inhibitors exhibited higher Michaelis–Menten constant (K_M_) values than those that did not. The increase in K_M_ means that the digestive proteases have a lower affinity for the substrate, so they require more substrate to work at half of their maximum speed (Vmax). These values illustrate the effect of competitive inhibitors, which compete with the substrate for binding to the enzyme's active site.^38^

In *S. frugiperda*, studies show that when exposed to Kunitz‐*like* inhibitors, the insect produces resistant enzyme variants with higher K_M_ values that are less affected by the inhibitor. In the study by Zhong *et al*.,^39^ new Kunitz‐*like* inhibitors, such as PeTI from *Platypodium elegans*, also exhibited higher K_M_ values. According to Ramalho *et al*.,^40^ these findings suggest that the increase in K_M_ indicates that the proteases are adapting or that the inhibitor is competing directly, which slows down protein breakdown and extends the larval stage, leading to poorer digestion and performance of the pest, a beneficial outcome for pest management.

When the larval weight of *S. frugiperda* was evaluated, a significant difference was observed in the caterpillars fed with an artificial diet containing the highest concentrations of the GORE 3 inhibitor, which were 0.04873%, 0.12216%, and 0.24432% (w/v), compared to caterpillars not fed with a diet containing the peptide. This suggests that inhibition of trypsin‐*like* proteases may impair protein digestion, leading to amino acid deficiency and reduced protein recycling, ultimately resulting in nutritional imbalance.^41^ A study conducted by Oliveira *et al*.^35^ had similar results. The caterpillars exposed to the tripeptide that has a lysine residue at position P1 became pupae with a lower body mass.

However, in the present study, there were no significant differences in pupal weight among individuals fed diets containing different tested concentrations of GORE 3. On the other hand, there were malformations in the pupae of *S. frugiperda* at the concentrations of 0.02436% and 0.04873% of GORE 3. These poorly formed pupae were unable to emerge as adults, thus disrupting their life cycles. Studies by Zhong *et al*.^39^ suggest that the ingestion or injection of specific inhibitors can lead to malformations, decrease pupation rates, and reduce adult emergence in *S. frugiperda*. For example, trehalase inhibitor compounds (ZK‐PI 5 and ZK‐PI 9) did not significantly alter the weight or length of the pupae. However, they reduced the pupation rate by up to 34.5% and the emergence rate by 34.7%, showing critical sublethal effects for pest control. In another study, sugar ester‐based compounds extracted from *Solanum sisymbriifolium* caused failure in the larva‐to‐pupa metamorphosis, resulting in mortality of up to 53% in the pupal stage, which indicated malformations during the metamorphic process.^42^

These effects may result from interference in energy metabolism or disruption of chitin formation, which are vital for the normal development of pupae.^43^ Such morphological changes support the potential of enzyme inhibitors as effective tools for strategies against *S. frugiperda*, even though they do not cause direct death, by impairing the crucial transition from larval to adult stages.

Furthermore, GORE 3 caused an extension of the larval period at all concentrations tested, regardless of the peptide. This effect increases larvae exposure to natural enemies and can reduce the *S. frugiperda* population in the field.^44^ Studies indicate that artificial diets containing enzyme inhibitors given to *S. frugiperda* often extend the larval stage, reflecting the larvae's efforts to overcome proteolytic inhibition.

In the study by Ramos *et al*.,^45^ the extract from the castor bean plant (*Ricinus communis*), rich in trypsin inhibitors, significantly extended the duration of the larval and pre‐pupal stages of the pest, while also decreasing pupal weight (at concentrations ≥909 μg mL^−1^). Another study, involving the expression of the BvSTI gene (beet serine protease inhibitor) in transgenic *Nicotiana benthamiana* plants, showed a delay of 1 to 3 days in insect pupation when fed the modified leaves, along with a reduction in weight gain; however, there was no significant increase in mortality.^46^

When examining the nutritional details, we observed a significant decrease in the approximate digestibility (AD%) of *S. frugiperda* caterpillars that consumed a diet containing 0.30% (w/v) of the GORE 3 inhibitor; this indicates they were less efficient at digesting the nutrients they ingested. This demonstrates that the peptide has a negative impact on protein digestion.

A similar trend was observed in studies with *S. frugiperda* that consumed diets containing Kunitz and Bowman‐Birk inhibitors, where the AD% was significantly lower than in the control group, linked to slower growth and an extended larval stage.^35^ Additionally, research by Hemmati *et al* ^47^ examined how a peptide from trypsinogen affected *Plodia interpunctella* larvae (Lepidoptera: Pyralidae) and found that the nutritional measures decreased due to the amount used.

The same concentrations tested for nutritional parameters were used to evaluate the leaf area index consumed. There was no significant difference in any of the concentrations. Despite the lack of significant differences in the parameters assessed after the foliar application of the inhibitor over a period of 4 h, these results may be related to factors such as exposure time, degradation of the inhibitor on the leaf surface, or inadequate ingestion of the bioactive compound by the caterpillars during this interval. Some studies have observed significant differences 24 h after applying inhibitors to corn or soybean leaves.^48^, ^49^, ^50^ Furthermore, formulations with surfactants or encapsulations are essential because they enhance the adherence and stability of the inhibitor on the leaf surface, allowing for substantially higher concentrations.

Our research group has previously investigated several peptides with inhibitory activity against insect digestive proteases. Taken together, these studies and the present results reinforce the relevance of protease‐inhibitor peptides as biological molecules capable of modulating digestive physiology in pest insects. In addition, research focused on their interactions with trypsin‐*like* enzymes in lepidopterans contributes to a more detailed understanding of the biochemical processes involved, which can support future efforts to refine and characterize these molecules.

## 5 CONCLUSION

Collectively, the molecular docking, enzymology, and bioassay data presented here demonstrate that GORE3 interacts specifically with *S. frugiperda* trypsin‐*like* enzymes and acts as a competitive inhibitor *in vitro*, achieving substantial inhibition and exhibiting conserved binding modes across digestive isoforms. These findings advance the biochemical characterization of this peptide and highlight its ability to engage multiple subsites within the active site cleft.

*In vivo*, GORE 3 induced measurable physiological effects, including changes in survival, digestive efficiency, and development, although such responses were accompanied by compensatory upregulation of gut proteases at higher exposures. These compensatory mechanisms limit the ability to conclude its practical suppressive potential and indicate that GORE3's biological effects occur within a complex physiological landscape.

Taken together, our results position GORE 3 as an informative model peptide, whose interaction with lepidopteran digestive proteases provides valuable insights into insect–peptidase dynamics. Future research focusing on stability, digestive resilience, and insect compensatory responses will be essential to determine whether derivatives or optimized formulations of such peptides could eventually be incorporated into broader studies on insect digestive physiology or plant–insect biochemical interactions.

## CONFLICT OF INTEREST

The authors confirm that there is no conflict of interest.

## AUTHOR CONTRIBUTIONS

**Daniel Guimarães Silva Paulo:** Conceptualization, data curation, data collection and investigation, formal analysis, funding acquisition, project administration, resources, software, visualization, writing — original draft, writing — review and editing. **Halina Schultz, Rafael Junior Andrade, Humberto Josué de Oliveira Ramos:** data collection and investigation, data curation, formal analysis, writing — original draft. **Ian Lucas Batista Santos, Maria Clara Neves Gomes Rodrigues, Milena Godoi Lima, Geisiane Aparecida Mariano, Yaremis Beatriz Meriño Cabrera:** data collection and investigation. **Maria Goreti de Almeida Oliveira:** conceptualization, data curation, formal analysis, funding acquisition, project administration, resources, software, supervision, validation, visualization, writing — original draft, writing — review and editing.

## Supporting information


**Data S1.** Supporting Information.

## Data Availability

The data that support the findings of this study are available from the corresponding author upon reasonable request.
