# Supplementary material for: Peptidic product derived from trypsin autolysis modulates insect digestive proteases and supports plant biochemical defense
Source: Pest Manag Sci. 2026 Jan 22;82(5):4632–47. doi: 10.1002/ps.70579 (PMC13071266; doi:10.1002/ps.70579)
Supplement: Supplementary file 1 — Data S1. Supporting Information. [file PS-82-4632-s001.pdf]

RAMACHANDRAN PLOT

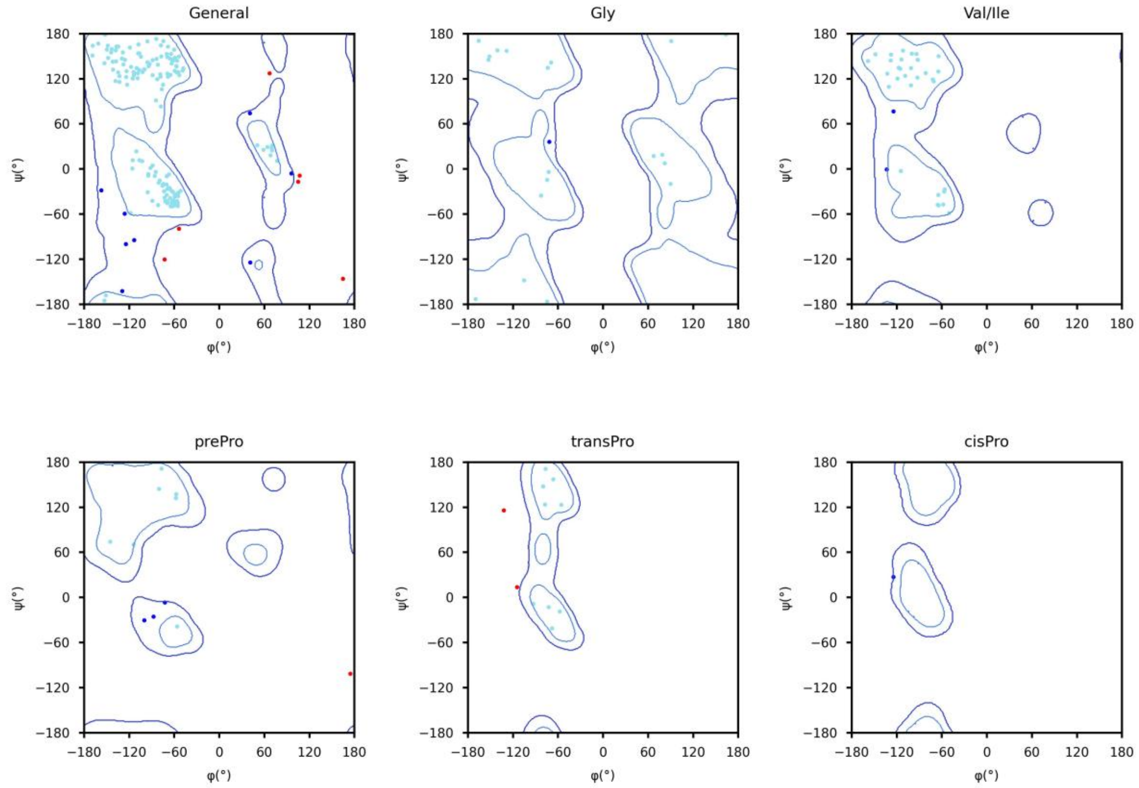

Spodoptera frugiperda 1:

Total		
Residue	239	
Ramachandran plot (Standard) statistics		
Favoured:	220	-92.05%
Allowed:	12	-5.02%
Disallowed:	7	-2.93%

RAMACHANDRAN PLOT

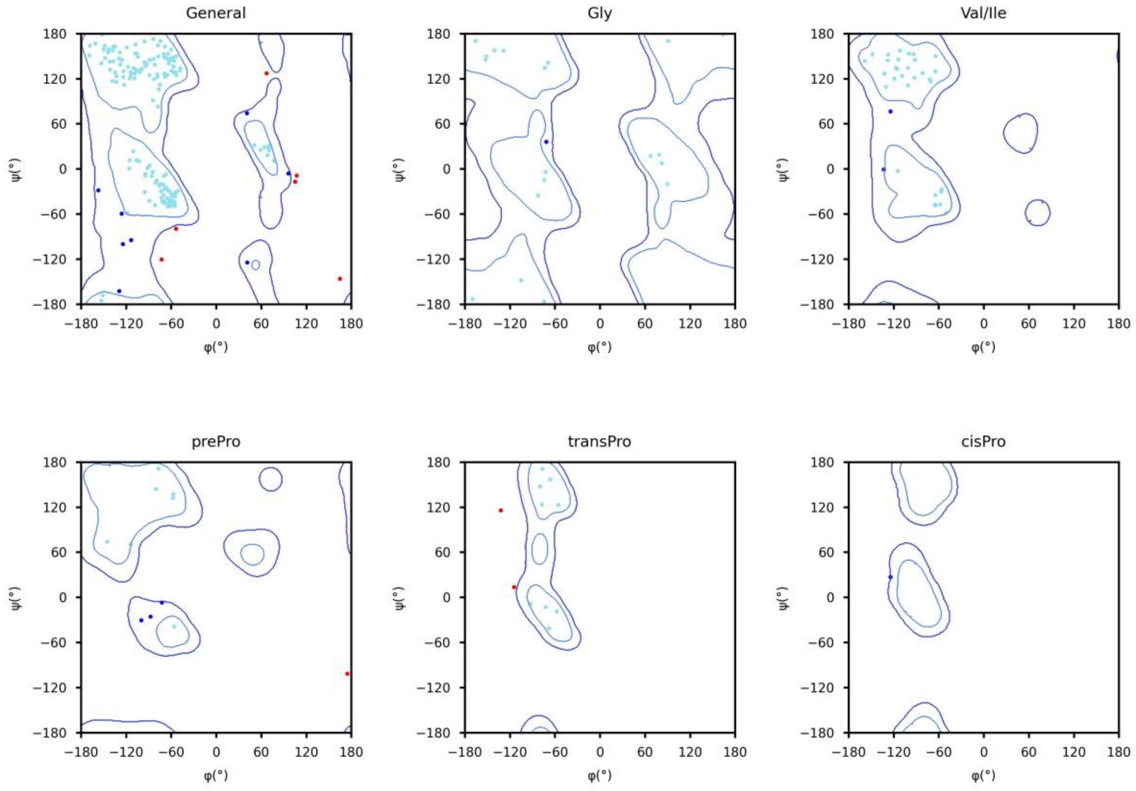

Spodoptera frugiperda 2:

Total		
Residue	225	
Ramachandran plot (Standard) statistics		
Favoured:	215	-95.56%
Allowed:	8	-3.56%
Disallowed:	2	-0.89%

RAMACHANDRAN PLOT

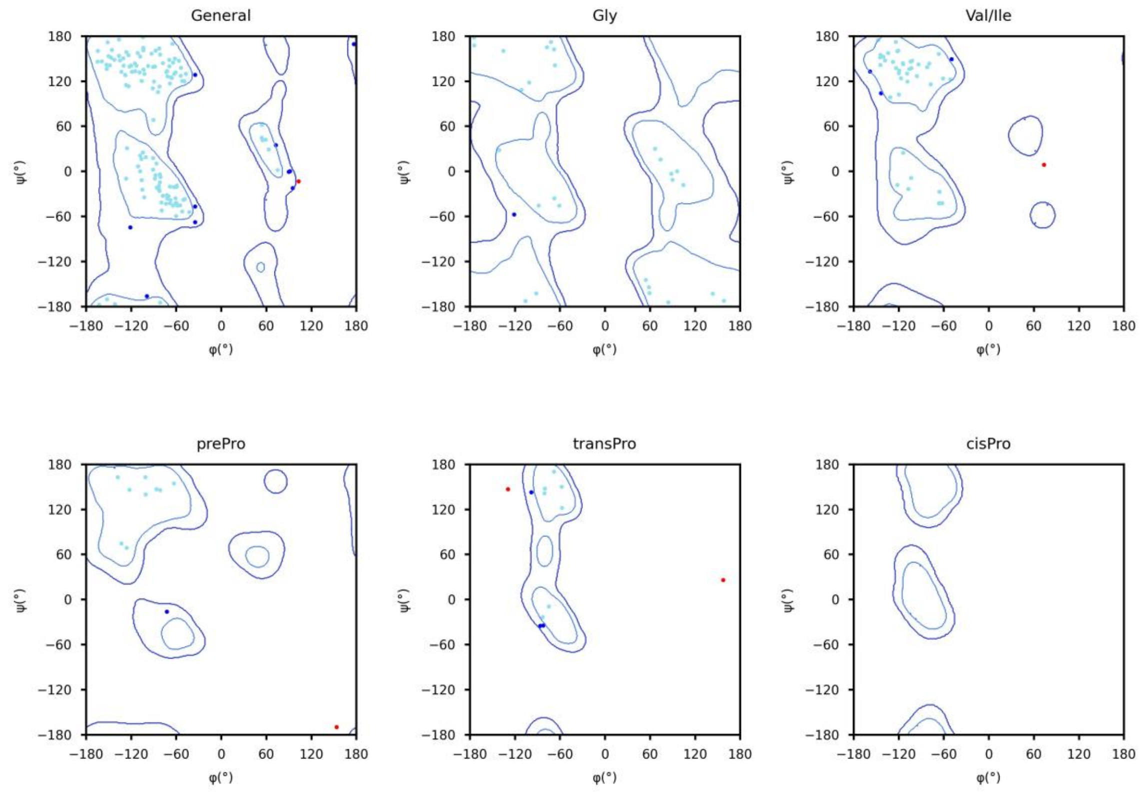

Spodoptera frugiperda 3

Total Residue	235	
Ramachandran plot (Standard) statistics		
Favoured:	219	-93.19%
Allowed:	13	-5.53%
Disallowed:	3	-1.28%

RAMACHANDRAN PLOT

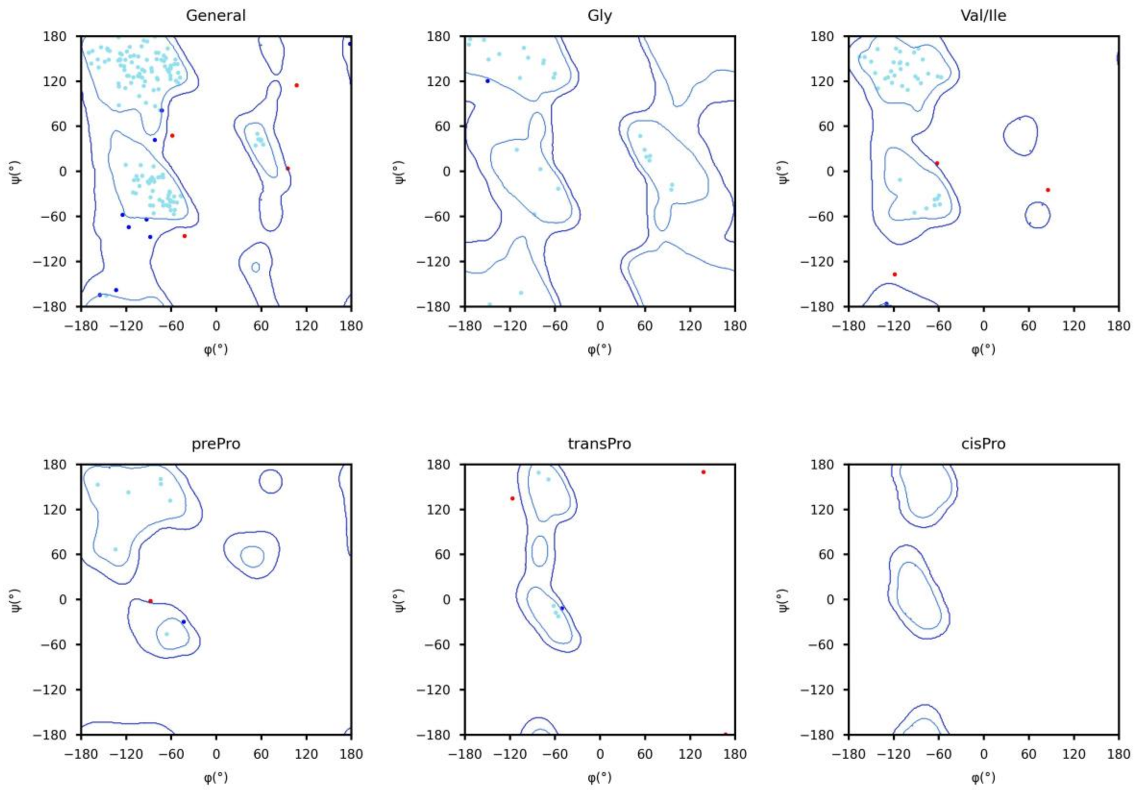

Spodoptera frugiperda 4

Total Residue	235	
Ramachandran plot (Standard) statistics		
Favoured:	213	-90.64%
Allowed:	15	-6.38%
Disallowed:	7	-2.98%
